# Tau deposition can commence in multiple cortical regions in Alzheimer's trajectory before coalescing into Braak Stages

**DOI:** 10.1002/alz70856_106752

**Published:** 2026-01-08

**Authors:** Rifa Sanjida Punnota, Roberto Vicidomini, Panagiotis Georgios Passias, Marie Emilie Tuil, Joseph Nowell, Paul Edison

**Affiliations:** ^1^ Imperial College London, London, Greater London, United Kingdom; ^2^ Imperial College London, London, London, United Kingdom; ^3^ Imperial College London, London, United Kingdom; ^4^ Cardiff University, Wales, United Kingdom

## Abstract

**Background:**

Tau pathology is a key hallmark of Alzheimer's Disease (AD), with its spread and regional deposition strongly correlated to cognitive decline. Braak staging describes a characteristic progression, beginning in the medial temporal lobe and extending to isocortical regions^1^. Here, we tested whether all individuals in the AD trajectory follow the classical pattern of Braak Staging and evaluated this using single‐subject voxel‐level evaluation of the tau deposition in a large participant cohort.

**Method:**

[^18^F]AV1451‐PET and T1‐weighted MRI (3T) scans were quantitatively analyzed for 362 participants from the ADNI database. Standardised Uptake Value Ratio (SUVR) images were computed, and single‐subject voxel‐wise analyses were performed by comparing individual scans to 50 amyloid‐negative Cognitively Normal (CN) controls generating ‘global’ measures from t‐statistical maps. Significant clusters of global tau deposition were visualized as 3D‐rendered maps, ranked by voxel count and sequentially arranged to depict tau distribution across AD and β‐amyloid‐positive (Aβ+) Mild Cognitive Impairment (MCI) and CN participants.

**Result:**

We observed that 65% of CN(Aβ+), 80% of MCI(Aβ+), and 90% of AD participants displayed tau distribution consistent with Braak Staging. However, we noticed that 35% of CN(Aβ+), 18% of MCI(Aβ+), and 11% of AD participants displayed tau deposition in smaller clusters in several cortical regions scattered across the brain, not following a fixed pattern. Notably, such distribution appeared within participants with <100,000 voxels affected (i.e. in very early stages). Participants with >100,000 voxels affected displayed tau distribution consistent with Braak Staging. The earliest stage, CN(Aβ+), had the highest percentage of participants displaying scattered spread.

**Conclusion:**

A pattern of tau spread, following Braak Staging, is likely to occur in later stages of the AD trajectory, whereas early on, there may be deposition in multiple cortical regions. It is possible that both the toxic insult leading to neuronal damage and tau deposition can occur in multiple cortical regions initially, but with disease progression, Braak regions likely become more vulnerable. These results may have significant implications in developing future tau‐targeted therapies and evaluation of efficacy of treatment.

**Reference**

1. Braak H et al. Staging Alzheimer's neurofibrillary pathology with immunocytochemistry. *Acta Neuropathol*. 2006;112(4):389–404.

